# Robotic Colonoscopy and Beyond: Insights into Modern Lower Gastrointestinal Endoscopy

**DOI:** 10.3390/diagnostics13142452

**Published:** 2023-07-23

**Authors:** Emanuele Tumino, Pierfrancesco Visaggi, Valeria Bolognesi, Linda Ceccarelli, Christian Lambiase, Sergio Coda, Purushothaman Premchand, Massimo Bellini, Nicola de Bortoli, Emanuele Marciano

**Affiliations:** 1 Endoscopy Unit, Azienda Ospedaliero Universitaria Pisana, 56125 Pisa, Italy; 2 Gastroenterology Unit, Department of Translational Research and New Technologies in Medicine and Surgery, University of Pisa, 56100 Pisa, Italy; 3 Digestive Disease Centre, Division of Surgery, Barking, Havering and Redbridge University Hospitals NHS Trust, Romford RM70AG, UK

**Keywords:** colonoscopy, robotic colonoscopy, lower gastrointestinal endoscopy

## Abstract

Lower gastrointestinal endoscopy is considered the gold standard for the diagnosis and removal of colonic polyps. Delays in colonoscopy following a positive fecal immunochemical test increase the likelihood of advanced adenomas and colorectal cancer (CRC) occurrence. However, patients may refuse to undergo conventional colonoscopy (CC) due to fear of possible risks and pain or discomfort. In this regard, patients undergoing CC frequently require sedation to better tolerate the procedure, increasing the risk of deep sedation or other complications related to sedation. Accordingly, the use of CC as a first-line screening strategy for CRC is hampered by patients’ reluctance due to its invasiveness and anxiety about possible discomfort. To overcome the limitations of CC and improve patients’ compliance, several studies have investigated the use of robotic colonoscopy (RC) both in experimental models and in vivo. Self-propelling robotic colonoscopes have proven to be promising thanks to their peculiar dexterity and adaptability to the shape of the lower gastrointestinal tract, allowing a virtually painless examination of the colon. In some instances, when alternatives to CC and RC are required, barium enema (BE), computed tomographic colonography (CTC), and colon capsule endoscopy (CCE) may be options. However, BE and CTC are limited by the need for subsequent investigations whenever suspicious lesions are found. In this narrative review, we discussed the current clinical applications of RC, CTC, and CCE, as well as the advantages and disadvantages of different endoscopic procedures, with a particular focus on RC.

## 1. Introduction

Colorectal cancer (CRC) is a major cause of neoplastic mortality, ranking third and second for worldwide cancer incidence and cancer-related deaths, respectively, leading to almost 1 million deaths per year [1,2]. Accordingly, in 2020, almost 2 million patients were diagnosed with CRC [1]. Most CRCs develop from preneoplastic colonic polyps, which can be present for years before the development of the CRC [3]. Accordingly, effective screening and early diagnosis can reduce mortality and improve outcomes in cancer patients [4,5,6]. For patients at risk of CRC, both invasive (i.e., colonoscopy) and non-invasive (i.e., fecal immunochemical testing, FIT) screening strategies are available [7]. When colonoscopy is delayed following a positive FIT, the likelihood of subsequent advanced adenomas, CRC, and advanced CRC increases [8]. In addition, it has been estimated that the mortality risk of CRC in patients not complying with a colonoscopy following a positive FIT is twice as high as that of patients who undergo a colonoscopy following a positive FIT [9]. Nevertheless, it has been estimated that 27.5% of FIT-positive patients do not undergo any subsequent lower gastrointestinal investigation [10]. Of note, lower gastrointestinal endoscopy is considered the gold standard for the diagnosis and removal of colonic polyps [11,12].

It is estimated that the removal of polyps is associated with a 60% reduction in CRC-related deaths [13] as a result of the reduction in the occurrence of the adenoma-carcinoma sequence [11]. In this regard, colonoscopy allows both the identification and removal of colonic polyps during a single procedure [14]. However, conventional colonoscopy (CC) is related to several potential procedural risks, including minor issues such as abdominal pain, abdominal distention, and bleeding [15], as well as severe complications such as cardio-pulmonary events [16,17], colon perforation [18,19], transmission of infections [20,21], and, rarely, death [22]. Overall, it has been determined that the incidence of complications in diagnostic colonoscopy ranges between 0.14% and 1.1% [23]. The colonoscope’s traction on the mesenteries during CC, which is done to solve loops or overcome angled colon tracts, is the main cause of abdominal pain. Additionally, the insufflation of air or carbon dioxide (CO_2_) used to stretch the colonic walls may cause discomfort and pain. In this regard, patients frequently require sedation to better tolerate CC, which may result in the onset of undesired deep sedation or complications related to sedation [24]. Accordingly, the use of CC as a first-line screening strategy for CRC is hampered by patients’ reluctance due to its invasiveness and anxiety about possible discomfort [25]. To face CC-related compliance issues, robotic colonoscopy (RC) was developed [26,27,28,29,30,31,32,33]. Differently from CC, RC systems generate internal forces and require minimal external pushing, which helps to limit pain and discomfort during the progression of the probe [31,32,33]. In this narrative review, we described technological advances in RC systems as well as the performance, advantages, and disadvantages of RC with the Endotics System (Era Endoscopy, Cascina, Pisa, Italy) compared to standard colonoscopy. 

## 2. Robotic Colonoscopy: What Is Available

RC systems represent the new frontier of endoscopic procedures, with an increasing number of scientific publications on the topic. These novel endoscopes are particularly promising thanks to their peculiar locomotion and adaptability to the shape of the lower gastrointestinal tract [34], which have been shown to provide benefits over CC both to patients and physicians. Accordingly, it has been shown that robotic colonoscopes can provide a more comfortable and less painful alternative to standard colonoscopy [30]. The only RC system that is currently available for use in clinical practice is the Endotics System, whose characteristics will be described below. 

### 2.1. The Endotics System (Era Endoscopy, Cascina, Pisa, Italy)

**Figure 1:**
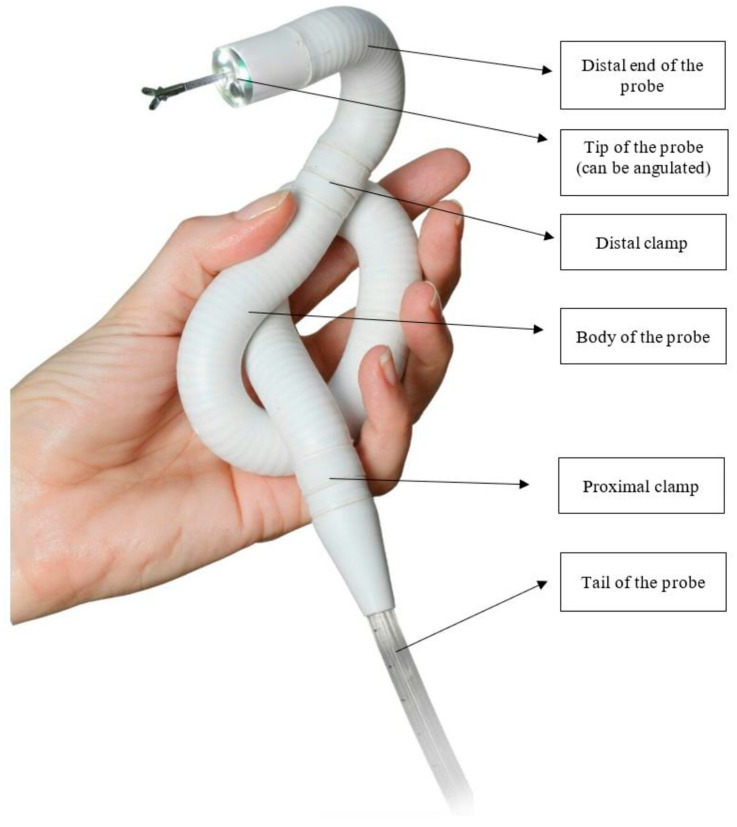
The probe of the Endotics System (image courtesy of Era Endoscopy S.R.L.).

**Figure 2:**
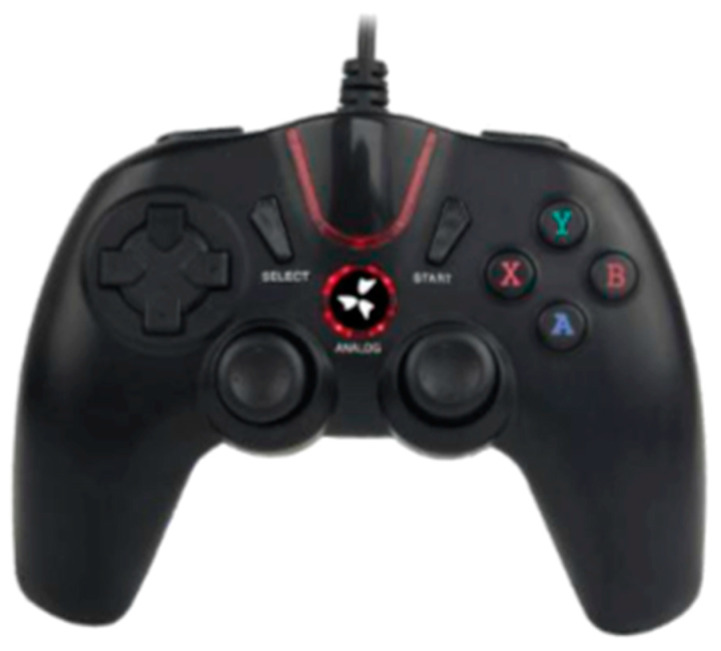
Control unit of the Endotics System (image courtesy of Era Endoscopy S.R.L.).

Endotics is an electro-pneumatic self-advancing locomotion RC system, which is currently the only available system in clinical practice (Figure 1) [31,32,33]. The device is controlled remotely by a hand-held control unit (Figure 2) and consists of a disposable colonoscope that advances in the colon using two mucosal clampers, located both proximally and distally on the probe (Figure 1). The Endotics System received the CE mark in 2017 and FDA 510(k) approval in 2020. The Endotics System is the only currently available robotic device in clinical practice. It is marketed in Europe, the UK, and Australia. The Endotics probe, also known as E-Worm, comes from the field of robotic biomimetic, a subfield of engineering research that examines the kinematic and dynamic systems of animals and plants with the aim of adapting them for use in the creation of robotic systems [31]. In particular, the Endotics System is inspired by the characteristic compass-like movement of a geometrid caterpillar. The probe is composed of a cranial end that can be oriented and has a rotation capability of 180 degrees in any direction. The body and tail are flexible and are connected to the workstation via a connector. The tail hosts electrical, pneumatic, and working channels, while the tip hosts the vision system, composed of a high-resolution camera with a viewing angle of 110 degrees, a light source, a channel for the water jet, and a channel for rinsing and suction/insufflation [33]. The body of the probe has a diameter of 17 mm and can vary its length: at rest, it is 25 cm long and reaches 43 cm when in maximum elongation. Repetitive movements of lengthening and shortening allow the locomotion of the probe. The movement is generated by a hydro-pneumatic system inside the probe body and by two clampers. One set of clamps is placed on the distal end of the body and another on the proximal end. The clampers adhere to the mucosa by suction. Both the length variations and the clampers are operated via the hydro-pneumatic system. The key operations performed by the Endotics System for locomotion are as follows: 1. the clamper on the proximal end of the E-Worm automatically adheres to the mucosa by suction; 2. the central part of the body of the E-Worm is elongated by the physician, who also steers the probe manually; 3. the clamper on the distal end on the E-Worm automatically adheres to the mucosa by suction; 4. the proximal clamper is automatically released; 5. the central part of the body is automatically contracted; 6. the clamper on the proximal end of the E-Worm automatically adheres to the mucosa by suction; 7. the distal clamp is automatically released; 8. the sequence starts over again. All the movements of the probe are controlled by a joystick held by the operator, who can steer the probe, elongate the body of the probe to move it forward, rinse, and use the working channels for insufflation and suction. 

### 2.2. Advantages of Robotic Colonoscopy with the Endotics System

The main advantage of the Endotics System is its peculiar locomotion activity, which reduces pressure on colonic walls during probe advancement and limits the perception of pain by patients. Ex vivo studies showed the Endotics System, compared to CC, exerted 90% lower pressure on the sensors around an experimental animal colon [31]. Data from a tandem study of 40 patients who underwent both CC and RC with the Endotics System showed that RC was significantly better tolerated than CC, with average pain and discomfort scores of 0.9 and 1.1 out of 10, respectively, compared to 6.9 and 6.8 for CC [33]. The reduction of pain perceived during RC compared to CC is also related to the fact that loops do not need to be disentangled by torsion, push, or pulling maneuvers. In this regard, using a hand-held console, the physician is able to elongate and steer the flexible probe following the shape of the bowel, even when narrow-angle loops are present [33]. Furthermore, it has been shown that the E-worm can adapt its shape to the configuration and curves of the colon, dramatically reducing the pain and discomfort related to colonic conformations that are difficult to negotiate [31]. Finally, due to its peculiar locomotion system that does not require pushing on the probe to advance, together with its high flexibility, the risk of colonic perforation is low [31,32,33] (Table 1).

### 2.3. Disadvantages of Robotic Colonoscopy with the Endotics System 

Older studies have shown that the Endotics System may require a higher degree of bowel cleansing compared to CC due to a small suction channel of 1 mm [33]. However, a novel model of the Endotics System with a 3 mm operative channel is now available, although data regarding operative procedures performed with this system are currently lacking. In general, adequate bowel preparation is crucial to the completion of the exam. During a lower endoscopy with the Endotics System, however, the presence of fecal residues can obstruct clampers and make it difficult for the clampers themselves to adequately adhere to the colic surface, compromising the automatic advancement of the probe. Accordingly, it has been shown that the number of colonoscopies that need to be prematurely terminated for poor bowel preparation is higher in RC than in CC [33]. Moreover, RC seems to have prolonged insertion times compared to CC [31,32]. Finally, pilot studies on small numbers of patients showed relatively lower cecal intubation rates (CIRs) than CC [31,32] (Table 1).

## 3. Robotic Colonoscopy: What Is Not Available

Other than the Endotics System, several other robotic flexible colonoscopes obtained CE marking or FDA 510 (k) approval (Table 2). However, most are not available for use in clinical practice as they have been withdrawn from the market. Here we report a summary of RC systems that have been manufactured in the past but are currently not available for use in clinical practice.

### 3.1. NeoGuide Endoscopy System (NeoGuide Endoscopy System Inc., Los Gatos, CA USA) 

This probe is propelled by electro-mechanical actuation with a “follow-the-leader” mechanism. It is composed of an insertion tube composed of 16 segments that controls the movement of the colonoscope [28]. Each segment is independent and has two degrees of freedom. The colonoscope has position sensors located at the distal end and at the external base that allow for a live view of the position of the scope’s tip, insertion depth, and computed real-time 3D mapping of the colon. Computerized mapping enables the insertion tube to change the segment shape at different insertion depths to reduce looping and unintentional lateral forces and, consequently, patient discomfort. This robotic endoscope obtained FDA approval in 2006. In 2007, Eickhoff and collaborators [28] published a prospective, nonrandomized, feasibility study conducted on 11 patients undergoing screening or diagnostic colonoscopy. The cecum was reached in all patients, who had high acceptance of the procedure. In addition, there were no adverse events at the last follow-up visit 30 days after the procedure. Despite such promising results, NeoGuide is no longer available on the market, and the technology is currently used for robotic-assisted minimally invasive peripheral lung biopsy.

### 3.2. Invendoscope—SC40 (Invendo Medical GmbH, Weinheim, Germany)

This robotic colonoscope has an electro-mechanical propulsion system with an inverted sleeve mechanism [29]. The colonoscope is moved forward and backward by an inverted-sleeve mechanism composed of eight wheels. In addition, the tip of the colonoscope is driven robotically, equipped with LEDs and a CMOS 114° camera, and electro-hydraulically flexed to 180° in any direction with full retroflection through a hand-held control unit. The diameter of the probe is 18 mm, and its working length is 2000 mm. Standard functions include suction, irrigation, and insufflation. The probe is also equipped with a 3.2 mm working channel, which can be used for conventional therapeutic procedures. In 2008, Rosch and colleagues [29] published a feasibility study on the use of the Invendoscope in 39 healthy volunteers. All procedures were performed without sedation, but instrument defects led to early termination of the procedures in five volunteers. In the remaining 34 procedures, the cecum was reached in 28 cases (82%), there were no reported adverse events, and discomfort scores were low [29]. The Invendoscope is no longer available on the market. 

### 3.3. Aer-O-Scope System (GI View Ltd., Ramat Gan, Israel)

This is a single-use, self-propelling, and self-steering colonoscope. The navigation happens through two inflatable balloons and internal pneumatic pressure (CO_2_) to push the frontal balloon forward and backward [35,36]. The colonoscope’s tip is teleoperated by a hand-held control unit and is accessorized with a 360° omni-directional HD vision system with a 57° FoV camera, LEDs, and two working channels. In addition, the system is equipped with electronic sensors for pressure monitoring. In 2006, Vucelic and colleagues [35] published a study investigating the feasibility of lower endoscopy with the Aer-O-Scope in 12 healthy volunteers. All examinations were conducted without sedation and were followed by CC for safety evaluation. The cecum was reached in 10 patients (83%), although two of these required analgesics and four experienced sweating and bloating. In 2016, the Aer-O-Scope received FDA approval. In the same year, Gluck et al. [36] published a study on the use of the Aer-O-Scope in 56 patients undergoing lower gastrointestinal endoscopy for CRC screening. The cecum was reached in 55 patients (98.2%) without the occurrence of mucosal damage or adverse events. However, the Aer-O-Scope only detected 87.5% of the polyps subsequently detected during a tandem CC. At present, GI View Ltd. no longer produces the self-propelling Aer-O-Scope. Instead, the technology is currently used on a single-use robotic colonoscope without the balloon propulsion.

### 3.4. ColonoSight (Stryker GI Ltd., Haifa, Israel)

This system consists of an electro-pneumatic self-advancing locomotion system, composed of a reusable colonoscope named EndoSight, which is equipped with LEDs and a camera and covered by a disposable multi-lumen sheath named ColonoSleeve [27]. The presence of a sleeve aims to eliminate the need for disinfection at the end of each procedure. The ColonoSight Model 510B received FDA approval in 2004. In 2008, Shike and colleagues published a study on the use of Colonosight in 19 animals and 178 patients [27]. The RC was conducted in all subjects without complications. In addition, the disposable parts of the endoscope allowed for the elimination of the need for the disinfection of the colonoscope between procedures, and the LED illumination eliminated the need for fiber optics and an external light source. The device is no longer manufactured at present. 

## 4. Robotic Colonoscopy in Current Clinical Practice

Studies on RC have been rapidly increasing in recent years. In 2017, Tumino et al. demonstrated that RC with the Endotics System was successfully performed in 93.1% of patients who had previously failed CC due to procedural pain [33]. In another study comparing the Endotics System with CC [32], although the cecum was reached in a significantly lower proportion of patients undergoing RC compared to CC and the average duration time of RC was significantly higher than that of CC, the two techniques had comparable ADR. However, of note, none of the patients undergoing RC required sedation, compared to 19.7% of patients undergoing CC. The authors also calculated the sensitivity and specificity of RC for polyps’ detection, which were 93.3% and 100%, respectively. In addition, 92.7% of patients were willing to have a repeat Endotics procedure. In another study, a painless RC was successfully performed in a patient with dolichocolon with severe angulations who refused to undergo CC with or without sedation due to the fear of perforation [34]. In another study, a direct comparison between RC with the Endotics System and CC was performed by Cosentino and colleagues [31]. RC had a significantly lower stress pattern on the colonic mucosa and higher diagnostic accuracy, possibly due to the lower insufflation of air that allowed for the visualization of small polyps and angiodysplasias not seen during CC. 

## 5. Non-Invasive and Minimally Invasive Alternatives to Conventional and Robotic Colonoscopy

There may be situations in which patients decline, cannot tolerate, or are unfit for both CC and RC. In such instances, the alternatives to investigating the lower gastrointestinal tract include barium enema (BE), computed tomographic colonography (CTC), and colon capsule endoscopy (CCE). CTC is currently strongly recommended as the radiological examination of choice for the diagnosis of colorectal neoplasia and is recommended for the diagnosis and follow-up when CC is contraindicated or not possible [37]. CCE is a disposable capsule that progresses through the colon and records color video footage. The device has two cameras that allow visualization of the colonic mucosa at 344 degrees [38]. Although CCE is not currently recommended as a first-line screening test for CRC, it can be used for CRC screening in patients with incomplete or unfeasible CC and a positive FIT [37]. In addition, some authors have proposed and investigated devices combining the use of robots with CCE [39,40]. Lucarini et al. [40] developed and tested ex vivo an external magnetic field locomotion system for colon capsules and demonstrated the feasibility of the system. Similarly, Verra et al. [39] published a study on the Endoo system in which a colon capsule, embedded with a magnet, is controlled externally by a magnet-equipped robot, which allows the navigation of the capsule in the colon. The Endoo system has been shown to have an ADR comparable to that of CC [39], but further studies are needed to further investigate the potential of robot-assisted CCE. However, the use of robots for the locomotion of colon capsules has the potential to further implement minimally invasive strategies for the direct visualization of the lower gastrointestinal tract. A recent randomized trial conducted on 5384 patients investigated the detection rate of CRC and polyps larger than 10 mm in patients undergoing BE and CTC or CC and CTC [41]. The authors found that CTC was superior to BE with regards to detection rates (7.3% vs. 5.6%), although there was no difference in the detection of CRC (3.7% vs. 3.4%). Of particular note, BE was found to have missed 14.1% of CRC compared to 6.7% for CTC, while CTC had a miss rate of 3.4% for CRC compared to 0% for CC at the three-year follow-up. In another randomized trial conducted in 21 centers in the UK [42], patients with symptoms suggestive of CRC were randomized to either CC (*n* = 1047) or CTC (*n* = 533). The detection rate of CRC was 11% for both CTC and CC. As many as 30% of patients undergoing CTC had additional colonic investigations, compared to 8.2% of those undergoing CC. However, around 50% of the referrals for subsequent investigations were for polyps smaller than 10 mm or because of clinical uncertainty, with a low predictive value for CRC. Of note, CTC missed 3.4% of CRC, while CC missed none. Moreover, in another recent randomized controlled trial [43], it was shown that CTC has suboptimal sensitivity for the detection of high-risk sessile serrated polyps, which are believed to be the precursors of up to 30% of all CRCs [44]. In particular, the detection rate of CTC was 0.8% compared to 4.3% for CC, demonstrating a markedly lower detection of high-risk lesions of CTC. 

A recent pilot study investigated the performance of CCE, CTC, and CC in 21 patients undergoing all three procedures in tandem [45]. The study showed that CC and CCE were comparable in terms of polyp detection, and both were superior to CTC. In addition, a recent systematic review with meta-analysis of 12 studies estimated a mean sensitivity, specificity, and diagnostic odds ratio of 0.85, 0.85, and 30.5, respectively, for the CCE for polyps of any size. However, the rates of complete CCE transit varied between 57% and 100%. Accordingly, the CCE seems to have suboptimal performance in the setting of CRC screening. In this regard, in a recent prospective randomized study, CCE was compared to CTC in a screening population of 286 individuals [46]. The study found that the proportion of patients with polyps of at least six millimeters confirmed by CC was 31.6% for CCE versus 8.6% for CTC, while for polyps of at least ten millimeters, the diagnostic yield was 13.5% with CCE versus 6.3% with CTC, thus showing that CCE was superior in the detection of polyps of at least six millimeters and non-inferior in the detection of polyps of at least ten millimeters compared to CTC. Another study investigated the use of CCE or CTC in patients with previous incomplete CC [47]. The study found that both CCE and CTC achieved colonic evaluation in 98% of cases, thus showing that CCE and CTC were comparable in terms of completing colon evaluation after incomplete colonoscopy. However, the study also showed that CCE had a significantly higher sensitivity compared to CTC, having detected 24.5% vs. 12.2% of polyps ≥ 6 mm. 

In terms of patients’ perspectives, a recent meta-analysis showed that patient preferences for CCE and CC were not significantly different [48]. However, although it has been recently shown that CCE is well tolerated, safe, and can reduce the proportion of patients requiring CC [49], another meta-analysis has demonstrated that both bowel cleanliness and complete transit of CCE are insufficient as compared to CC [50]. In addition, it must be acknowledged that a systematic review has demonstrated that the inter/intra-observer agreement of capsule endoscopy is suboptimal, possibly due to heterogeneity in Country, capsule type, and bowel cleansing scale among included studies [51]. In this regard, it has been shown that the use of prucalopride can increase the proportion of patients with complete colon capsule transit and acceptable preparation [52]. Similarly, in a recent study, sulfate-based bowel preparations were superior to polyethylene glycol in terms of bowel cleansing in patients undergoing CCE [53]. 

## 6. Discussion and Conclusions

Endoscopy represents the gold standard for the diagnosis and removal of colonic polyps [11,12]. However, it is not infrequent that patients decline CC, even following a positive FIT, due to potential risks and fear of pain [10]. The colonoscope’s traction on the mesenteries is the main cause of abdominal pain during CC. In addition, the insufflation of air or CO2 during CC may cause discomfort and pain. Accordingly, sedation may be required during CC, which may result in the onset of unintended deep sedation or complications related to sedation [24]. When CC takes longer than ten minutes to reach the caecum, it is defined as long lasting colonoscopy (LLC). LLC represents another factor that may increase patient discomfort during CC. In this regard, a RCT is currently recruiting patients to investigate the safety and efficacy of Endorail in improving CC completion rates in LLC (NCT05626738). Endorail is a CC add-on device that works as a magnetic anchor to guide the colonoscope and to straighten colon curves and loops. When the caecal intubation time is longer than 10 min, the balloon catheter is inserted in the tool channel, advanced beyond the colonoscope tip, and filled with Ferromagnetic Fluid. The Endorail Handpiece is then applied over the patient’s abdomen to anchor the balloon. The anchored balloon guide allows for straightening the scope and the colon itself. The colonoscope can thus be easily moved back and forth along the anchored guide to facilitate colonoscope positioning and colonoscopy completion. Afterward, the Endorail is removed, and the straightened colonoscope can be easily pushed forward to achieve colonoscopy completion according to standard endoscopic technique.

In recent years, the use of robots and artificial intelligence in clinical practice has increased considerably [6,54]. Accordingly, several studies have investigated the use of RC as an alternative to CC. In particular, it has been shown that RC may be better tolerated by patients who failed a previous CC due to pain [33]. In addition, it has been shown that patients undergoing RC require sedation in a significantly lower proportion of cases [32]. Accordingly, a study by Cosentino et al. showed that the stress pattern on the colonic mucosa related to RC was 90% lower than that of CC. Another possible advantage of RC over CC is that the insertion phase already allows adequate visualization of the colonic mucosa, while the withdrawal phase provides the opportunity to double-check what has already been seen during insertion. In contrast, the diagnostic phase of CC can only be performed during the withdrawal phase. Accordingly, it has been shown that RC may have higher diagnostic accuracy compared to CC due to the lower insufflation rate, which may allow for visualization of small lesions not seen during the standard colonoscopy. However, it must be noted that both RC and CC have comparable ADR [32], although the time required to complete a RC is usually longer [31,32]. With regards to artificial intelligence-aided endoscopy, it has been recently shown that the use of computer-aided diagnosis (CAD) systems is cost-effective when used in fecal immunochemical test-positive patients compared to CC [55]. In addition, CAD systems have shown accuracy in predicting the invasion depth of early CRC [56] and in improving the ADR during CC [57]. Accordingly, position statements from international endoscopy Societies are becoming available, paving the way for the systematic use of CAD systems in routine clinical practice [58,59].

Alternative diagnostic techniques such as CTC and CCE may be required when CC and RC are contraindicated or unavailable. These diagnostic tests have been shown to be safe and well tolerated by patients [48,49]. However, although a number of studies demonstrated the good accuracy of the tests in terms of polyp detection [48,60], international guidelines currently restrict the use of CTC and CCE to very selected cases [37]. CTC is suitable for CRC screening in locations where there is no organized FIT-based population CRC program, and both CTC and CCE are recommended as subsequent investigations following positive FIT in patients with incomplete or unfeasible colonoscopy [37]. It must be stressed, however, that whenever suspicious lesions are found at the CTC or CCE, subsequent more invasive investigations are required. 

In conclusion, the higher tolerability of RC may enhance the use of lower gastrointestinal endoscopy as a primary screening strategy for CRC, possibly improving patients’ compliance. Alternatives to CC and RC include BE, CTC, and CCE, which may be suitable in selected cases, according to the clinical scenario. More prospective and possibly randomized clinical trials are required to consolidate knowledge of RC and implement the use of this modern endoscopic procedure in clinical practice. 

## Figures and Tables

**Table 1:** Reports of advantages and disadvantages of RC with the Endotics System.

Advantages	Disadvantages
Reduced pressure on colonic walls during advancement	Requires an adequate cleaning to achieve completion of the exam (superior to CC)
Reduction in pain compared to CC (0.9/10 vs. 6.9/10)	Prolonged insertion time and prolonged procedure time
Reduction in discomfort compared to CC (1.1/10 vs. 6.8/10)	No data regarding operative procedures with Endotics System
Useful in patients who failed CC because of reported pain (alternative to CC in sedation)	Relatively lower CIR (small pilot studies)
Rapid learning curve	
Drastically reduction in colonic perforation	
ADR comparable with CC	

Abbreviations. ADR—Adenoma Detection Rate; CIR—Cecal Intubation rate; CC—conventional colonoscopy.

**Table 2:** Critical attributes of robotic colonoscopy systems.

Robotic Colonoscopy System	Characteristics	Availability on the Market
**Endotics System**	Electro-pneumatic self-advancing locomotion The device is controlled remotely by a hand-held control unit	AVAILABLE in clinical practice
**NeoGuide Endoscopy System**	Electro-mechanical propulsion with a “follow-the-leader” mechanism. Composed by a 16-segment insertion tube that controls the snake-like movement of the probe. Position sensors are located at the distal tip and at the external base of the device to obtain live view of the position of the scope, insertion depth, and computed real-time 3D mapping of the colon	NOT AVAILABLE
**Invendoscope SC40**	Electro-mechanical propulsion with an inverted sleeve mechanism Has a robotically driven tip controlled remotely by a hand-held control unit	NOT AVAILABLE
**Aer-O-Scope System**	Self-propelling, self-steering, and disposable. The locomotion happens through two inflatable balloons (distal and proximal end of the probe) and internal pneumatic pressure for pushing the frontal mobile balloon forward and backward.	NOT AVAILABLE
**ColonoSight System**	Electro-mechanical propulsion Has a reusable part (the colonoscope) and a disposable part (multi-lumen sheath with working channel.	NOT AVAILABLE
**Robotic-assisted Colonoscopy Capsule**	Magnetic colon capsule with an external magnetic field locomotion system An external robot with a magnet is used to navigate the capsule in the colon.	AVAILABLE for experimental use

## Data Availability

Data sharing not applicable.
